# Supplementary material for: Evaluation of Polyphenol Content and Antioxidant Capacity of Aqueous Extracts from Eight Medicinal Plants from Reunion Island: Protection against Oxidative Stress in Red Blood Cells and Preadipocytes
Source: Antioxidants (Basel). 2020 Oct 7;9(10):959. doi: 10.3390/antiox9100959 (PMC7650546; doi:10.3390/antiox9100959)
Supplement: Supplementary file 1 [file antioxidants-09-00959-s001.docx]

**SUPPLEMENTARY DATA**

**Table 1 – SUPPLEMENTARY DATA: Reported bioactivity of the 8 selected medicinal plants**

| *Aphloia theiformis* |
| --- |
| *Aphloia theiformis* belongs to the aphloiaceae family; its leaves, barks and roots are widely used as herbal beverage. This plant is found in East Africa, Madagascar, Mascarene and Seychelles areas. *Aphloia theiformis* was traditionally known and used for treatment of digestive disorders in young children [1], treatment of fever, stomach pain and albuminuria [2], for blood pressure lowering [3], as a dewormer [4] and for its photoprotective properties [5]. In the context of metabolic diseases, *A. theiformis* is used for its antidiabetic and cholesterol lowering properties [6]. |
| *Ayapana triplinervis* |
| *Ayapana triplinervis* is a well-known asteraceae from Brazil. Also known as *Eupatorium triplinerve,* this plant was traditionally used in India in case of pain and inflammatory disorders [7,8]. In Mauritius Island, this plant is usually prepared as a decoction for children stomach pain, diarrhoea, colitis and vomiting symptoms [9]. Introduced in Reunion Island in the 1800s, herbal beverages with *Ayapana triplinervis* leaves were used for their antiemetic properties and protective effects against dysentery and digestive problems. The essential oil of *A. triplinervis* and its major component the thymohydroquinone dimethyl ether was recently reported to exert anti-viral activity against Zika virus. [10,11] |
| *Dodonaea viscosa* |
| This indigenous plant of Reunion Island can be found in subtropical regions of South Africa, South America, Asia and Oceania. Traditionally used as depurative, beverage against renal colic, kidney stones, osteoarthritis, gout and rheumatism, this plant showed interesting antimicrobial, antioxidant, anti-inflammatory, analgesic and anti-ulcer activity [12]. In the context of metabolic disorders, the use of *D viscosa* was found to have antispasmodic, antidiarrheal and antidiabetic actions [6]. |
| *Hubertia ambavilla* |
| This endemic asteraceae of Reunion Island is traditionally used to improve blood circulation, to prevent diabetes, eczema, wounds, rheumatism, itch, asthma and ulcer. It has been described for its antioxidant properties [13,14]. |
| *Hypericum lanceolatum* |
| This endemic hypericaceae of Reunion Island is traditionally prepared for preventing fever, cystitis, bad blood circulation and dysmenorrhoea but also for its antioxidant and antimicrobial activities [14–16]. |
| *Pelargonium x graveolens* |
| This plant is commonly used for its antioxidant, haemostatic, anti-inflammatory anti-eczema, and anti-rheumatism activities but also as analgesic and skin astringent. This geraniaceae rich in essential oil is also known to have antidiabetic and antioxidant properties [17,18]. |
| *Psiloxylon mauritianum* |
| This endemic psiloxylaceae can only be found in Reunion and Mauritius Islands. Known for its contents in asiatic and corosolic acids, this plant was firstly described as a treatment for amenorrhea and dysentery with diuretic, anti-inflammatory, depurative and cholesterol-lowering properties [2]. |
| *Syzygium cumini* |
| This plant is probably the most studied of our selection; it belongs to the myrtaceae family and is used in Brazil, Reunion Island, India and Asia as an antidiabetic agent and has been described to exert antioxidant activity.[19,20] |

**Table 2 – SUPPLEMENTARY DATA: Reported bioactivity of the main polyphenols detected in medicinal plant polyphenol rich extracts (PRE), infusions and decoctions.**

| **Component** | **Plant** | **Bioactivity** |
| --- | --- | --- |
| Mangiferin | *Aphloia theiformis* | Prevents disorders in skin regeneration [21]  Action on key enzymes associated with type 2 diabetes complications [22]  Inhibition of metabolic enzymes (acetylcholine esterase, cholesterol esterase, glycogen phosphorylase a) and angiotensin (1) converting enzyme [22]  Antioxidant capacity [23,24]  Cardioprotective effects [25]  Impairs Zika virus attachment to host cell surface[10]  Anti-chikungunya activity [26] |
| Aphloiol |  | Prevent red blood cell lysis [2,27] |
| Caffeoyl glucarate | *Ayapana triplinervis, Pelargonium x graveolens, Dodonaea viscosa* | Modulation of pro-inflammatory response to LPS [28] |
| Coumarin derivatives Ayapin | *Ayapana triplinervis* | Antinociceptive, antioxidant effects [8] |
| Herniarin |  | Protection against DNA damages [29], anti-inflammatory activity [30], anti-cancer [31] |
| Thymohydroquinone dimethyl ether |  | Inhibition of zika virus [11] |
| Quercetin | *Ayapana triplinervis, Dodonaea viscosa, Hubertia ambavilla, Pelargonium x graveolens, Psiloxylon mauritianum,,Hypericum lanceolatum* | Antihypertensive effects [32], anti-obesity effects [33], neuroprotective effects and antioxidant activity [34], antidiabetic effects [35], cardiovascular protective effects [36], chemopreventive potential [37], anti-inflammatory effects [38] |
| Chlorogenic acid | *Dodonaea viscosa, Ayapana triplinervis, Hubertia ambavilla* | Antioxidant activity [39], Anti-inflammatory effects [40], Anti-obesity, improves lipid metabolism [41] |
| Procyanidins | *Hypericum lanceolatum, Dodonaea viscosa* | Inhibitory effects on α-amylase [42], anti-inflammatory activity by down-regulating macrophage polarization [43], insulin sensitizer in adipocytes [44], reduces liver fibrosis, over-deposition of extracellular matrix and angiogenesis [45], modulation of gut microbiota and impact on Non Alcoholic Fatty Liver Disease and Diet induced-obesity [46] |
| Catechin | *Dodonaea viscosa* | Protection against oxidative stress [47], prevention  of infectious diseases [48], antioxidant activity [49,50] |
| Epicatechin | *Dodonaea viscosa* | Antioxidant activity [50], reduces blood pressure [51], protects beta cell activity [52], protective effects on steatosis and insulin resistance [53] |
| Isorhamnetin | *Hypericum lanceolatum, Dodonaea viscosa* | Protection against cardiac hypertrophy [54], ameliorates metabolic disorders [55], hepatoprotective effects [56], anti-inflammatory effects against LPS stimulation [57], prevention of oxidative stress induced by H_2_O_2_ [58] |
| Coumaroyl quinic acid | *Dodonaea viscosa* | Antioxidant activity [55], anti-inflammatory effects by inhibition of NFκB [59], peroxynitrite-scavenging effects [60] |
| Kaempferol | *Psiloxylon mauritianum, Pelargonium x graveolens* | Antioxidant activity [61,62], anti-inflammatory properties [63], cardiovascular protective effects [64–66], prevent red blood cell lysis induced by Reactive Oxygen Species [67], anti-cancer action [68] |
| Myricetin | *Pelargonium x graveolens* | Cardioprotective effects [69], anti-inflammatory activity [70,71], antidiabetic activity [72], protection against metabolic disorders [73] |
| Gallic acid | *Syzygium cumini* | Antioxidant and neuroprotective agent [74], anti-inflammatory effects [75],  impact on metabolism and insulin resistance [76,77], protection against oxidative stress [78] |
| Vescalagin |  | Potential in chemotherapy [79,80], action on insulin resistance [81], down regulation of lipid profile and glycemia [82] |
| Castalagin |  | Effects on osteoclastogenesis [83] |
| Galloyl tannins |  | α-glucosidase inhibitory activity, cholesterol lowering [84] |
| Ellagic acid |  | Antioxidant, anti-inflammatory effects [85–87], cardioprotective effects [88,89], protection against oxidative stress [11] |
| HHDP (hexahydroxydiphenic acid) |  | Anti-inflammatory activity [90] moderate lipase inhibition activity [91] |
| Caffeic acid | *Hypericum lanceolatum* | Reduces oxidative stress [92–94] metabolic effects on hepatic steatosis [95], anti-inflammatory effects [96,97] |
| Asiatic acid | *Psiloxylon mauritianum* | Anti-inflammatory effects [26,98–100], protective effects against oxidative stress damage [101–103], cardioprotective effects [42,104] |
| Caffeoyl quinic acid | *Hypericum lanceolatum, Hubertia ambavilla* | Antioxidant activity [105,106] |

**Figure 1 SUPPLEMENTARY DATA: Comparative study of the protective effect of medicinal plant infusions and decoctions at 25 µM GAE final concentration in response to H_2_O_2_.** 3T3-L1 preadipocytes were incubated with medicinal plant infusions and decoctions. DCF fluorescence was measured at 492 nm for excitation and 520 nm for emission. Data shown are means ± SEM of three independent experiments ### p<0.005 as compared to control (PBS alone, or H_2_O_2_)_._

**References**

1. Duchemann, B. *“Les plantes médicinales” Exposition universelle de 1900. Colonies et protectorats.*; **1900**;

2. Lavergne, R. Plantes médicinales indigènes: tisanerie et tisaneurs de la Réunion, Université Montpellier II - Sciences et Techniques du Languedoc: France, **1989**.

3. Lopez, J.-G.; Drouet, G.; de Haro, L. Hypotension sévère au cours d’une intoxication par deux plantes médicinales à l’île de la Réunion : *Aphloia theiformis* et *Rubus alceifolius*. *Annales de Toxicologie Analytique* **2013**, *25*, 121–123, doi:10.1051/ata/2013046.

4. Decary, R. Plantes utiles de la Flore Malgache. *Revue internationale de botanique appliquée et d’agriculture tropicale* **1979**, 338–339.

5. Danthu, P.; Lubrano, C.; Flavet, L.; Rahajanirina, V.; Behra, O.; Fromageot, C.; Rabevohitra, R.; Roger, E. Biological Factors Influencing Production of Xanthones in *Aphloia theiformis*. *Chemistry & Biodiversity* **2010**, *7*, 140–150, doi:10.1002/cbdv.200900199.

6. Mootoosamy, A.; Fawzi Mahomoodally, M. Ethnomedicinal application of native remedies used against diabetes and related complications in Mauritius. *Journal of Ethnopharmacology* **2014**, *151*, 413–444, doi:10.1016/j.jep.2013.10.069.

7. Cheriyan, B.V.; Kadhirvelu, P.; Nadipelly, J.; Shanmugasundaram, J.; Sayeli, V.; Subramanian, V. Anti-nociceptive Effect of 7-methoxy Coumarin from Eupatorium Triplinerve vahl (Asteraceae). *Pharmacogn Mag* **2017**, *13*, 81–84, doi:10.4103/0973-1296.197650.

8. Melo, A.S.; Monteiro, M.C.; da Silva, J.B.; de Oliveira, F.R.; Vieira, J.L.F.; de Andrade, M.A.; Baetas, A.C.; Sakai, J.T.; Ferreira, F.A.; Cunha Sousa, P.J. da; et al. Antinociceptive, neurobehavioral and antioxidant effects of Eupatorium triplinerve Vahl on rats. *Journal of Ethnopharmacology* **2013**, *147*, 293–301, doi:10.1016/j.jep.2013.03.002.

9. Mahomoodally, M.F.; Sreekeesoon, D.P. A Quantitative Ethnopharmacological Documentation of Natural Pharmacological Agents Used by Pediatric Patients in Mauritius. *Biomed Res Int* **2014**, *2014*, doi:10.1155/2014/136757.

10. Clain, E.; Sinigaglia, L.; Koishi, A.C.; Gorgette, O.; Gadea, G.; Viranaicken, W.; Krejbich-Trotot, P.; Mavingui, P.; Desprès, P.; Nunes Duarte dos Santos, C.; et al. Extract from Aphloia theiformis, an edible indigenous plant from Reunion Island, impairs Zika virus attachment to the host cell surface. *Sci Rep* **2018**, *8*, doi:10.1038/s41598-018-29183-2.

11. Haddad, J.G.; Picard, M.; Bénard, S.; Desvignes, C.; Desprès, P.; Diotel, N.; El Kalamouni, C. Ayapana triplinervis Essential Oil and Its Main Component Thymohydroquinone Dimethyl Ether Inhibit Zika Virus at Doses Devoid of Toxicity in Zebrafish. *Molecules* **2019**, *24*, doi:10.3390/molecules24193447.

12. Arun, M.; Asha, V.V. Gastroprotective effect of Dodonaea viscosa on various experimental ulcer models. *Journal of Ethnopharmacology* **2008**, *118*, 460–465, doi:10.1016/j.jep.2008.05.026.

13. Ben Haddada, M.; Gerometta, E.; Chawech, R.; Sorres, J.; Bialecki, A.; Pesnel, S.; Spadavecchia, J.; Morel, A.-L. Assessment of antioxidant and dermoprotective activities of gold nanoparticles as safe cosmetic ingredient. *Colloids Surf B Biointerfaces* **2020**, *189*, 110855, doi:10.1016/j.colsurfb.2020.110855.

14. Poullain, C.; Girard-Valenciennes, E.; Smadja, J. Plants from reunion island: evaluation of their free radical scavenging and antioxidant activities. *Journal of Ethnopharmacology* **2004**, *95*, 19–26, doi:10.1016/j.jep.2004.05.023.

15. Couladis, M.; Baziou, P.; Verykokidou, E.; Loukis, A. Antioxidant activity of polyphenols from Hypericum triquetrifolium Turra. *Phytotherapy Research* **2002**, *16*, 769–770, doi:10.1002/ptr.1062.

16. Tchakam, P.D.; Lunga, P.K.; Kowa, T.K.; Lonfouo, A.H.N.; Wabo, H.K.; Tapondjou, L.A.; Tane, P.; Kuiate, J.-R. Antimicrobial and antioxidant activities of the extracts and compounds from the leaves of Psorospermum aurantiacum Engl. and Hypericum lanceolatum Lam. *BMC Complementary and Alternative Medicine* **2012**, *12*, doi:10.1186/1472-6882-12-136.

17. Boukhris, M.; Bouaziz, M.; Feki, I.; Jemai, H.; El Feki, A.; Sayadi, S. Hypoglycemic and antioxidant effects of leaf essential oil of Pelargonium graveolens L’Hér. in alloxan induced diabetic rats. *Lipids in Health and Disease* **2012**, *11*, 81, doi:10.1186/1476-511X-11-81.

18. Lavergne, R. *Les plantes médicinales réunionnaises d’aujourd’hui -*; 2018;

19. Ayyanar, M.; Subash-Babu, P.; Ignacimuthu, S. Syzygium cumini (L.) Skeels., a novel therapeutic agent for diabetes: Folk medicinal and pharmacological evidences. *Complementary Therapies in Medicine* **2013**, *21*, 232–243, doi:10.1016/j.ctim.2013.03.004.

20. dos Santos, M.M.; Prestes, A.S.; de Macedo, G.T.; Ecker, A.; Barcelos, R.P.; Boligon, A.A.; Souza, D.; de Bem, A.F.; da Rocha, J.B.T.; Barbosa, N.V. Syzygium cumini leaf extract inhibits LDL oxidation, but does not protect the liproprotein from glycation. *Journal of Ethnopharmacology* **2018**, *210*, 69–79, doi:10.1016/j.jep.2017.08.033.

21. Pleguezuelos-Villa, M.; Nácher, A.; Hernández, M.J.; Ofelia Vila Buso, M.A.; Ruiz Sauri, A.; Díez-Sales, O. Mangiferin nanoemulsions in treatment of inflammatory disorders and skin regeneration. *Int J Pharm* **2019**, *564*, 299–307, doi:10.1016/j.ijpharm.2019.04.056.

22. Picot, M.C.N.; Bender, O.; Atalay, A.; Zengin, G.; Loffredo, L.; Hadji-Minaglou, F.; Mahomoodally, M.F. Multiple pharmacological targets, cytotoxicity, and phytochemical profile of Aphloia theiformis (Vahl.) Benn. *Biomedicine & Pharmacotherapy* **2017**, *89*, 342–350, doi:10.1016/j.biopha.2017.02.031.

23. Dembitsky, V.M.; Poovarodom, S.; Leontowicz, H.; Leontowicz, M.; Vearasilp, S.; Trakhtenberg, S.; Gorinstein, S. The multiple nutrition properties of some exotic fruits: Biological activity and active metabolites. *Food Research International* **2011**, *44*, 1671–1701, doi:10.1016/j.foodres.2011.03.003.

24. Lim, Y.P.; Pang, S.F.; Yusoff, M.M.; Abdul Mudalip, S.K.; Gimbun, J. Correlation between the extraction yield of mangiferin to the antioxidant activity, total phenolic and total flavonoid content of Phaleria macrocarpa fruits. *Journal of Applied Research on Medicinal and Aromatic Plants* **2019**, *14*, 100224, doi:10.1016/j.jarmap.2019.100224.

25. Jiang, T.; Han, F.; Gao, G.; Liu, M. Mangiferin exert cardioprotective and anti-apoptotic effects in heart failure induced rats. *Life Sciences* **2020**, *249*, 117476, doi:10.1016/j.lfs.2020.117476.

26. Ledoux, A.; Cao, M.; Jansen, O.; Mamede, L.; Campos, P.-E.; Payet, B.; Clerc, P.; Grondin, I.; Girard-Valenciennes, E.; Hermann, T.; et al. Antiplasmodial, anti-chikungunya virus and antioxidant activities of 64 endemic plants from the Mascarene Islands. *Int. J. Antimicrob. Agents* **2018**, *52*, 622–628, doi:10.1016/j.ijantimicag.2018.07.017.

27. Jonville, M.C.; Kodja, H.; Strasberg, D.; Pichette, A.; Ollivier, E.; Frédérich, M.; Angenot, L.; Legault, J. Antiplasmodial, anti-inflammatory and cytotoxic activities of various plant extracts from the Mascarene Archipelago. *Journal of Ethnopharmacology* **2011**, *136*, 525–531, doi:10.1016/j.jep.2010.06.013.

28. Dudek, M.K.; Michalak, B.; Woźniak, M.; Czerwińska, M.E.; Filipek, A.; Granica, S.; Kiss, A.K. Hydroxycinnamoyl derivatives and secoiridoid glycoside derivatives from Syringa vulgaris flowers and their effects on the pro-inflammatory responses of human neutrophils. *Fitoterapia* **2017**, *121*, 194–205, doi:10.1016/j.fitote.2017.07.008.

29. Rezaee, R.; Behravan, E.; Behravan, J.; Soltani, F.; Naderi, Y.; Emami, B.; Iranshahi, M. Antigenotoxic activities of the natural dietary coumarins umbelliferone, herniarin and 7-isopentenyloxy coumarin on human lymphocytes exposed to oxidative stress. *Drug Chem Toxicol* **2014**, *37*, 144–148, doi:10.3109/01480545.2013.834352.

30. Nayeli, M.-B.; Maribel, H.-R.; Enrique, J.-F.; Rafael, B.-P.; Margarita, A.-F.; Macrina, F.-M.; Ivan, M.-D.; Manasés, G.-C. Anti-inflammatory activity of coumarins isolated from Tagetes lucida Cav. *Nat. Prod. Res.* **2019**, 1–5, doi:10.1080/14786419.2018.1553172.

31. Kielbus, M.; Skalicka-Wozniak, K.; Grabarska, A.; Jeleniewicz, W.; Dmoszynska-Graniczka, M.; Marston, A.; Polberg, K.; Gawda, P.; Klatka, J.; Stepulak, A. 7-substituted coumarins inhibit proliferation and migration of laryngeal cancer cells in vitro. *Anticancer Res.* **2013**, *33*, 4347–4356.

32. Marunaka, Y.; Marunaka, R.; Sun, H.; Yamamoto, T.; Kanamura, N.; Inui, T.; Taruno, A. Actions of Quercetin, a Polyphenol, on Blood Pressure. *Molecules* **2017**, *22*, doi:10.3390/molecules22020209.

33. Ahn, J.; Lee, H.; Kim, S.; Park, J.; Ha, T. The anti-obesity effect of quercetin is mediated by the AMPK and MAPK signaling pathways. *Biochemical and Biophysical Research Communications* **2008**, *373*, 545–549, doi:10.1016/j.bbrc.2008.06.077.

34. Costa, L.G.; Garrick, J.M.; Roquè, P.J.; Pellacani, C. Mechanisms of Neuroprotection by Quercetin: Counteracting Oxidative Stress and More. *Oxidative Medicine and Cellular Longevity* **2016**, *2016*, 1–10, doi:10.1155/2016/2986796.

35. Eid, H.M.; Haddad, P.S. The Antidiabetic Potential of Quercetin: Underlying Mechanisms. *Curr. Med. Chem.* **2017**, *24*, 355–364, doi:10.2174/0929867323666160909153707.

36. Patel, R.V.; Mistry, B.M.; Shinde, S.K.; Syed, R.; Singh, V.; Shin, H.-S. Therapeutic potential of quercetin as a cardiovascular agent. *European Journal of Medicinal Chemistry* **2018**, *155*, 889–904, doi:10.1016/j.ejmech.2018.06.053.

37. Kashyap, D.; Garg, V.K.; Tuli, H.S.; Yerer, M.B.; Sak, K.; Sharma, A.K.; Kumar, M.; Aggarwal, V.; Sandhu, S.S. Fisetin and Quercetin: Promising Flavonoids with Chemopreventive Potential. *Biomolecules* **2019**, *9*, doi:10.3390/biom9050174.

38. Carullo, G.; Cappello, A.R.; Frattaruolo, L.; Badolato, M.; Armentano, B.; Aiello, F. Quercetin and derivatives: useful tools in inflammation and pain management. *Future Med Chem* **2017**, *9*, 79–93, doi:10.4155/fmc-2016-0186.

39. Tošović, J.; Marković, S.; Dimitrić Marković, J.M.; Mojović, M.; Milenković, D. Antioxidative mechanisms in chlorogenic acid. *Food Chem* **2017**, *237*, 390–398, doi:10.1016/j.foodchem.2017.05.080.

40. Naveed, M.; Hejazi, V.; Abbas, M.; Kamboh, A.A.; Khan, G.J.; Shumzaid, M.; Ahmad, F.; Babazadeh, D.; FangFang, X.; Modarresi-Ghazani, F.; et al. Chlorogenic acid (CGA): A pharmacological review and call for further research. *Biomedicine & Pharmacotherapy* **2018**, *97*, 67–74, doi:10.1016/j.biopha.2017.10.064.

41. Cho, A.-S.; Jeon, S.-M.; Kim, M.-J.; Yeo, J.; Seo, K.-I.; Choi, M.-S.; Lee, M.-K. Chlorogenic acid exhibits anti-obesity property and improves lipid metabolism in high-fat diet-induced-obese mice. *Food and Chemical Toxicology* **2010**, *48*, 937–943, doi:10.1016/j.fct.2010.01.003.

42. Dai, T.; Chen, J.; Li, Q.; Li, P.; Hu, P.; Liu, C.; Li, T. Investigation the interaction between procyanidin dimer and α-amylase: Spectroscopic analyses and molecular docking simulation. *Int. J. Biol. Macromol.* **2018**, *113*, 427–433, doi:10.1016/j.ijbiomac.2018.01.189.

43. Tian, Y.; Yang, C.; Yao, Q.; Qian, L.; Liu, J.; Xie, X.; Ma, W.; Nie, X.; Lai, B.; Xiao, L.; et al. Procyanidin B2 Activates PPARγ to Induce M2 Polarization in Mouse Macrophages. *Front Immunol* **2019**, *10*, 1895, doi:10.3389/fimmu.2019.01895.

44. Sun, P.; Li, K.; Wang, T.; Ji, J.; Wang, Y.; Chen, K.-X.; Jia, Q.; Li, Y.-M.; Wang, H.-Y. Procyanidin C1, a Component of Cinnamon Extracts, Is a Potential Insulin Sensitizer That Targets Adipocytes. *J. Agric. Food Chem.* **2019**, *67*, 8839–8846, doi:10.1021/acs.jafc.9b02932.

45. Feng, J.; Wang, C.; Liu, T.; Li, J.; Wu, L.; Yu, Q.; Li, S.; Zhou, Y.; Zhang, J.; Chen, J.; et al. Procyanidin B2 inhibits the activation of hepatic stellate cells and angiogenesis via the Hedgehog pathway during liver fibrosis. *J. Cell. Mol. Med.* **2019**, *23*, 6479–6493, doi:10.1111/jcmm.14543.

46. Xing, Y.-W.; Lei, G.-T.; Wu, Q.-H.; Jiang, Y.; Huang, M.-X. Procyanidin B2 protects against diet-induced obesity and non-alcoholic fatty liver disease via the modulation of the gut microbiota in rabbits. *World J. Gastroenterol.* **2019**, *25*, 955–966, doi:10.3748/wjg.v25.i8.955.

47. Bernatoniene, J.; Kopustinskiene, D.M. The Role of Catechins in Cellular Responses to Oxidative Stress. *Molecules* **2018**, *23*, doi:10.3390/molecules23040965.

48. Reygaert, W.C. Green Tea Catechins: Their Use in Treating and Preventing Infectious Diseases. *Biomed Res Int* **2018**, *2018*, 9105261, doi:10.1155/2018/9105261.

49. Pandey, K.B.; Rizvi, S.I. Plant Polyphenols as Dietary Antioxidants in Human Health and Disease. *Oxidative Medicine and Cellular Longevity* **2009**, *2*, 270–278, doi:10.4161/oxim.2.5.9498.

50. Grzesik, M.; Naparło, K.; Bartosz, G.; Sadowska-Bartosz, I. Antioxidant properties of catechins: Comparison with other antioxidants. *Food Chemistry* **2018**, *241*, 480–492, doi:10.1016/j.foodchem.2017.08.117.

51. Ellinger, S.; Reusch, A.; Stehle, P.; Helfrich, H.-P. Epicatechin ingested via cocoa products reduces blood pressure in humans: a nonlinear regression model with a Bayesian approach. *Am. J. Clin. Nutr.* **2012**, *95*, 1365–1377, doi:10.3945/ajcn.111.029330.

52. Martín, M.Á.; Fernández-Millán, E.; Ramos, S.; Bravo, L.; Goya, L. Cocoa flavonoid epicatechin protects pancreatic beta cell viability and function against oxidative stress. *Molecular Nutrition & Food Research* **2014**, *58*, 447–456, doi:10.1002/mnfr.201300291.

53. Cremonini, E.; Wang, Z.; Bettaieb, A.; Adamo, A.M.; Daveri, E.; Mills, D.A.; Kalanetra, K.M.; Haj, F.G.; Karakas, S.; Oteiza, P.I. (-)-Epicatechin protects the intestinal barrier from high fat diet-induced permeabilization: Implications for steatosis and insulin resistance. *Redox Biol* **2018**, *14*, 588–599, doi:10.1016/j.redox.2017.11.002.

54. Gao, L.; Yao, R.; Liu, Y.; Wang, Z.; Huang, Z.; Du, B.; Zhang, D.; Wu, L.; Xiao, L.; Zhang, Y. Isorhamnetin protects against cardiac hypertrophy through blocking PI3K-AKT pathway. *Mol. Cell. Biochem.* **2017**, *429*, 167–177, doi:10.1007/s11010-017-2944-x.

55. Zhang, Y.; Gu, M.; Cai, W.; Yu, L.; Feng, L.; Zhang, L.; Zang, Q.; Wang, Y.; Wang, D.; Chen, H.; et al. Dietary component isorhamnetin is a PPARγ antagonist and ameliorates metabolic disorders induced by diet or leptin deficiency. *Scientific Reports* **2016**, *6*, doi:10.1038/srep19288.

56. Lu, X.; Liu, T.; Chen, K.; Xia, Y.; Dai, W.; Xu, S.; Xu, L.; Wang, F.; Wu, L.; Li, J.; et al. Isorhamnetin: A hepatoprotective flavonoid inhibits apoptosis and autophagy via P38/PPAR-α pathway in mice. *Biomed. Pharmacother.* **2018**, *103*, 800–811, doi:10.1016/j.biopha.2018.04.016.

57. Kim, S.Y.; Jin, C.-Y.; Kim, C.H.; Yoo, Y.H.; Choi, S.H.; Kim, G.-Y.; Yoon, H.M.; Park, H.T.; Choi, Y.H. Isorhamnetin alleviates lipopolysaccharide-induced inflammatory responses in BV2 microglia by inactivating NF-κB, blocking the TLR4 pathway and reducing ROS generation. *Int. J. Mol. Med.* **2019**, *43*, 682–692, doi:10.3892/ijmm.2018.3993.

58. Wang, J.; Gong, H.-M.; Zou, H.-H.; Liang, L.; Wu, X.-Y. Isorhamnetin prevents H2O2‑induced oxidative stress in human retinal pigment epithelial cells. *Mol Med Rep* **2018**, *17*, 648–652, doi:10.3892/mmr.2017.7916.

59. Ahn, J.H.; Park, Y.; Jo, Y.H.; Kim, S.B.; Yeon, S.W.; Kim, J.G.; Turk, A.; Song, J.Y.; Kim, Y.; Hwang, B.Y.; et al. Organic acid conjugated phenolic compounds of hardy kiwifruit (Actinidia arguta) and their NF-κB inhibitory activity. *Food Chem* **2020**, *308*, 125666, doi:10.1016/j.foodchem.2019.125666.

60. Nugroho, A.; Kim, K.H.; Lee, K.R.; Alam, M.B.; Choi, J.S.; Kim, W.-B.; Park, H.-J. Qualitative and quantitative determination of the caffeoylquinic acids on the Korean mountainous vegetables used for chwinamul and their peroxynitrite-scavenging effect. *Arch. Pharm. Res.* **2009**, *32*, 1361–1367, doi:10.1007/s12272-009-2003-6.

61. Park, J.-W.; Choi, J.-S.; Choi, J.-S. Effects of Kaempferol, an Antioxidant, on the Bioavailability and Pharmacokinetics of Nimodipine in Rats. *Journal of Pharmaceutical Investigation* **2011**, *41*, 301–307, doi:10.4333/KPS.2011.41.5.301.

62. Teffo, L.S.; Aderogba, M.A.; Eloff, J.N. Antibacterial and antioxidant activities of four kaempferol methyl ethers isolated from Dodonaea viscosa Jacq. var. angustifolia leaf extracts. *South African Journal of Botany* **2010**, *76*, 25–29, doi:10.1016/j.sajb.2009.06.010.

63. Devi, K.P.; Malar, D.S.; Nabavi, S.F.; Sureda, A.; Xiao, J.; Nabavi, S.M.; Daglia, M. Kaempferol and inflammation: From chemistry to medicine. *Pharmacol. Res.* **2015**, *99*, 1–10, doi:10.1016/j.phrs.2015.05.002.

64. Dabeek, W.M.; Marra, M.V. Dietary Quercetin and Kaempferol: Bioavailability and Potential Cardiovascular-Related Bioactivity in Humans. *Nutrients* **2019**, *11*, doi:10.3390/nu11102288.

65. Kong, L.; Luo, C.; Li, X.; Zhou, Y.; He, H. The anti-inflammatory effect of kaempferol on early atherosclerosis in high cholesterol fed rabbits. *Lipids Health Dis* **2013**, *12*, 115, doi:10.1186/1476-511X-12-115.

66. Zhong, X.; Zhang, L.; Li, Y.; Li, P.; Li, J.; Cheng, G. Kaempferol alleviates ox-LDL-induced apoptosis by up-regulation of miR-26a-5p via inhibiting TLR4/NF-κB pathway in human endothelial cells. *Biomed. Pharmacother.* **2018**, *108*, 1783–1789, doi:10.1016/j.biopha.2018.09.175.

67. Wu, P.; Meng, X.; Zheng, H.; Zeng, Q.; Chen, T.; Wang, W.; Zhang, X.; Su, J. Kaempferol Attenuates ROS-Induced Hemolysis and the Molecular Mechanism of Its Induction of Apoptosis on Bladder Cancer. *Molecules* **2018**, *23*, doi:10.3390/molecules23102592.

68. Wang, X.; Yang, Y.; An, Y.; Fang, G. The mechanism of anticancer action and potential clinical use of kaempferol in the treatment of breast cancer. *Biomed. Pharmacother.* **2019**, *117*, 109086, doi:10.1016/j.biopha.2019.109086.

69. Zhang, N.; Feng, H.; Liao, H.-H.; Chen, S.; Yang, Z.; Deng, W.; Tang, Q.-Z. Myricetin attenuated LPS induced cardiac injury in vivo and in vitro. *Phytother Res* **2018**, *32*, 459–470, doi:10.1002/ptr.5989.

70. Hou, W.; Hu, S.; Su, Z.; Wang, Q.; Meng, G.; Guo, T.; Zhang, J.; Gao, P. Myricetin attenuates LPS-induced inflammation in RAW 264.7 macrophages and mouse models. *Future Med Chem* **2018**, *10*, 2253–2264, doi:10.4155/fmc-2018-0172.

71. Xie, J.; Zheng, Y. Myricetin protects keratinocyte damage induced by UV through IκB/NFκb signaling pathway. *J Cosmet Dermatol* **2017**, *16*, 444–449, doi:10.1111/jocd.12399.

72. Li, Y.; Zheng, X.; Yi, X.; Liu, C.; Kong, D.; Zhang, J.; Gong, M. Myricetin: a potent approach for the treatment of type 2 diabetes as a natural class B GPCR agonist. *FASEB J.* **2017**, *31*, 2603–2611, doi:10.1096/fj.201601339R.

73. Xia, S.-F.; Le, G.-W.; Wang, P.; Qiu, Y.-Y.; Jiang, Y.-Y.; Tang, X. Regressive Effect of Myricetin on Hepatic Steatosis in Mice Fed a High-Fat Diet. *Nutrients* **2016**, *8*, doi:10.3390/nu8120799.

74. Yen, G.-C.; Duh, P.-D.; Tsai, H.-L. Antioxidant and pro-oxidant properties of ascorbic acid and gallic acid. *Food Chemistry* **2002**, *79*, 307–313, doi:10.1016/S0308-8146(02)00145-0.

75. BenSaad, L.A.; Kim, K.H.; Quah, C.C.; Kim, W.R.; Shahimi, M. Anti-inflammatory potential of ellagic acid, gallic acid and punicalagin A&B isolated from Punica granatum. *BMC Complement Altern Med* **2017**, *17*, 47, doi:10.1186/s12906-017-1555-0.

76. Abdel-Moneim, A.; El-Twab, S.M.A.; Yousef, A.I.; Reheim, E.S.A.; Ashour, M.B. Modulation of hyperglycemia and dyslipidemia in experimental type 2 diabetes by gallic acid and p -coumaric acid: The role of adipocytokines and PPARγ. *Biomedicine & Pharmacotherapy* **2018**, *105*, 1091–1097, doi:10.1016/j.biopha.2018.06.096.

77. Oboh, G.; Ogunsuyi, O.B.; Ogunbadejo, M.D.; Adefegha, S.A. Influence of gallic acid on α-amylase and α-glucosidase inhibitory properties of acarbose. *Journal of Food and Drug Analysis* **2016**, *24*, 627–634, doi:10.1016/j.jfda.2016.03.003.

78. Dludla, P.V.; Nkambule, B.B.; Jack, B.; Mkandla, Z.; Mutize, T.; Silvestri, S.; Orlando, P.; Tiano, L.; Louw, J.; Mazibuko-Mbeje, S.E. Inflammation and Oxidative Stress in an Obese State and the Protective Effects of Gallic Acid. *Nutrients* **2018**, *11*, doi:10.3390/nu11010023.

79. Auzanneau, C.; Montaudon, D.; Jacquet, R.; Elkaoukabi-Chaibi, A.; Quideau, S.; De Giorgi, F.; Ichas, F.; Pourquier, P. R5: La vescalagine, ellagitanin polyphénolique, est un inhibiteur spécifique de l’isoforme alpha de la topo-isomérase 2 humaine. *Bulletin du Cancer* **2010**, *97*, S18–S19, doi:10.1016/S0007-4551(15)30922-X.

80. Kamada, Y.; Yakabu, H.; Ichiba, T.; Tamanaha, A.; Shimoji, M.; Kato, M.; Norimoto, C.; Yamashiro, R.; Miyagi, I.; Sakudo, A.; et al. Castalagin and vescalagin purified from leaves of Syzygium samarangense (Blume) Merrill & L.M. Perry: Dual inhibitory activity against PARP1 and DNA topoisomerase II. *Fitoterapia* **2018**, *129*, 94–101, doi:10.1016/j.fitote.2018.06.015.

81. Huang, D.-W.; Chang, W.-C.; Wu, J.S.-B.; Shih, R.-W.; Shen, S.-C. Vescalagin from Pink Wax Apple [Syzygium samarangense (Blume) Merrill and Perry] Alleviates Hepatic Insulin Resistance and Ameliorates Glycemic Metabolism Abnormality in Rats Fed a High-Fructose Diet. *J. Agric. Food Chem.* **2016**, *64*, 1122–1129, doi:10.1021/acs.jafc.5b05558.

82. Shen, S.-C.; Chang, W.-C. Hypotriglyceridemic and hypoglycemic effects of vescalagin from Pink wax apple [Syzygium samarangense (Blume) Merrill and Perry cv. Pink] in high-fructose diet-induced diabetic rats. *Food Chem* **2013**, *136*, 858–863, doi:10.1016/j.foodchem.2012.08.037.

83. Iwatake, M.; Okamoto, K.; Tanaka, T.; Tsukuba, T. Castalagin Exerts Inhibitory Effects on Osteoclastogenesis Through Blocking a Broad Range of Signaling Pathways with Low Cytotoxicity. *Phytother Res* **2015**, *29*, 917–924, doi:10.1002/ptr.5333.

84. Gato, N.; Kadowaki, A.; Hashimoto, N.; Yokoyama, S.; Matsumoto, K. Persimmon Fruit Tannin-Rich Fiber Reduces Cholesterol Levels in Humans. *Annals of Nutrition and Metabolism* **2013**, *62*, 1–6, doi:10.1159/000343787.

85. Boehning, A.L.; Essien, S.A.; Underwood, E.L.; Dash, P.K.; Boehning, D. Cell type-dependent effects of ellagic acid on cellular metabolism. *Biomedicine & Pharmacotherapy* **2018**, *106*, 411–418, doi:10.1016/j.biopha.2018.06.142.

86. Corbett, S.; Daniel, J.; Drayton, R.; Field, M.; Steinhardt, R.; Garrett, N. Evaluation of the Anti-inflammatory Effects of Ellagic Acid. *Journal of PeriAnesthesia Nursing* **2010**, *25*, 214–220, doi:https://doi.org/10.1016/j.jopan.2010.05.011.

87. Derosa, G.; Maffioli, P.; Sahebkar, A. Ellagic Acid and Its Role in Chronic Diseases. *Adv. Exp. Med. Biol.* **2016**, *928*, 473–479, doi:10.1007/978-3-319-41334-1_20.

88. Long, L.; Song, Y. Dietary ellagic acid is protective for atherosclerosis. *International Journal of Cardiology* **2018**, *256*, 12, doi:10.1016/j.ijcard.2017.12.094.

89. Mele, L.; Mena, P.; Piemontese, A.; Marino, V.; López-Gutiérrez, N.; Bernini, F.; Brighenti, F.; Zanotti, I.; Del Rio, D. Antiatherogenic effects of ellagic acid and urolithins in vitro. *Archives of Biochemistry and Biophysics* **2016**, *599*, 42–50, doi:10.1016/j.abb.2016.02.017.

90. Pinheiro, A.J.M.C.R.; Mendes, A.R.S.; Neves, M.D.F. de J.; Prado, C.M.; Bittencourt-Mernak, M.I.; Santana, F.P.R.; Lago, J.H.G.; de Sá, J.C.; da Rocha, C.Q.; de Sousa, E.M.; et al. Galloyl-Hexahydroxydiphenoyl (HHDP)-Glucose Isolated From Punica granatum L. Leaves Protects Against Lipopolysaccharide (LPS)-Induced Acute Lung Injury in BALB/c Mice. *Front Immunol* **2019**, *10*, 1978, doi:10.3389/fimmu.2019.01978.

91. Huang, Y.-L.; Tsujita, T.; Tanaka, T.; Matsuo, Y.; Kouno, I.; Li, D.-P.; Nonaka, G. Triterpene hexahydroxydiphenoyl esters and a quinic acid purpurogallin carbonyl ester from the leaves of Castanopsis fissa. *Phytochemistry* **2011**, *72*, 2006–2014, doi:10.1016/j.phytochem.2011.07.007.

92. Koga, M.; Nakagawa, S.; Kato, A.; Kusumi, I. Caffeic acid reduces oxidative stress and microglial activation in the mouse hippocampus. *Tissue Cell* **2019**, *60*, 14–20, doi:10.1016/j.tice.2019.07.006.

93. Pittalà, V.; Salerno, L.; Romeo, G.; Acquaviva, R.; Di Giacomo, C.; Sorrenti, V. Therapeutic Potential of Caffeic Acid Phenethyl Ester (CAPE) in Diabetes. *Curr. Med. Chem.* **2018**, *25*, 4827–4836, doi:10.2174/0929867324666161118120908.

94. Sato, Y.; Itagaki, S.; Kurokawa, T.; Ogura, J.; Kobayashi, M.; Hirano, T.; Sugawara, M.; Iseki, K. In vitro and in vivo antioxidant properties of chlorogenic acid and caffeic acid. *Int J Pharm* **2011**, *403*, 136–138, doi:10.1016/j.ijpharm.2010.09.035.

95. Kim, H.M.; Kim, Y.; Lee, E.S.; Huh, J.H.; Chung, C.H. Caffeic acid ameliorates hepatic steatosis and reduces ER stress in high fat diet–induced obese mice by regulating autophagy. *Nutrition* **2018**, *55–56*, 63–70, doi:10.1016/j.nut.2018.03.010.

96. Basu Mallik, S.; Mudgal, J.; Nampoothiri, M.; Hall, S.; Dukie, S.A.-; Grant, G.; Rao, C.M.; Arora, D. Caffeic acid attenuates lipopolysaccharide-induced sickness behaviour and neuroinflammation in mice. *Neurosci. Lett.* **2016**, *632*, 218–223, doi:10.1016/j.neulet.2016.08.044.

97. Choi, H.G.; Tran, P.T.; Lee, J.-H.; Min, B.S.; Kim, J.A. Anti-inflammatory activity of caffeic acid derivatives isolated from the roots of Salvia miltiorrhiza Bunge. *Arch. Pharm. Res.* **2018**, *41*, 64–70, doi:10.1007/s12272-017-0983-1.

98. Fong, L.Y.; Ng, C.T.; Yong, Y.K.; Hakim, M.N.; Ahmad, Z. Asiatic acid stabilizes cytoskeletal proteins and prevents TNF-α-induced disorganization of cell-cell junctions in human aortic endothelial cells. *Vascular Pharmacology* **2019**, *117*, 15–26, doi:10.1016/j.vph.2018.08.005.

99. Hao, C.; Wu, B.; Hou, Z.; Xie, Q.; Liao, T.; Wang, T.; Ma, D. Asiatic acid inhibits LPS-induced inflammatory response in human gingival fibroblasts. *Int. Immunopharmacol.* **2017**, *50*, 313–318, doi:10.1016/j.intimp.2017.07.005.

100. Qian, Y.; Xin, Z.; Lv, Y.; Wang, Z.; Zuo, L.; Huang, X.; Li, Y.; Xin, H.-B. Asiatic acid suppresses neuroinflammation in BV2 microglia via modulation of the Sirt1/NF-κB signaling pathway. *Food Funct* **2018**, *9*, 1048–1057, doi:10.1039/c7fo01442b.

101. Loganathan, C.; Thayumanavan, P. Asiatic acid prevents the quinolinic acid-induced oxidative stress and cognitive impairment. *Metab Brain Dis* **2018**, *33*, 151–159, doi:10.1007/s11011-017-0143-9.

102. Qi, Z.; Ci, X.; Huang, J.; Liu, Q.; Yu, Q.; Zhou, J.; Deng, X. Asiatic acid enhances Nrf2 signaling to protect HepG2 cells from oxidative damage through Akt and ERK activation. *Biomedicine & Pharmacotherapy* **2017**, *88*, 252–259, doi:10.1016/j.biopha.2017.01.067.

103. Ramachandran, V.; Saravanan, R. Asiatic acid prevents lipid peroxidation and improves antioxidant status in rats with streptozotocin-induced diabetes. *Journal of Functional Foods* **2013**, *5*, 1077–1087, doi:10.1016/j.jff.2013.03.003.

104. Bunbupha, S.; Prachaney, P.; Kukongviriyapan, U.; Kukongviriyapan, V.; Welbat, J.U.; Pakdeechote, P. Asiatic acid alleviates cardiovascular remodelling in rats with L-NAME-induced hypertension. *Clin. Exp. Pharmacol. Physiol.* **2015**, *42*, 1189–1197, doi:10.1111/1440-1681.12472.

105. Hung, T.M.; Na, M.; Thuong, P.T.; Su, N.D.; Sok, D.; Song, K.S.; Seong, Y.H.; Bae, K. Antioxidant activity of caffeoyl quinic acid derivatives from the roots of Dipsacus asper Wall. *J Ethnopharmacol* **2006**, *108*, 188–192, doi:10.1016/j.jep.2006.04.029.

106. Hwang, Y.P.; Yun, H.J.; Chun, H.K.; Chung, Y.C.; Kim, H.K.; Jeong, M.H.; Yoon, T.R.; Jeong, H.G. Protective mechanisms of 3-caffeoyl, 4-dihydrocaffeoyl quinic acid from Salicornia herbacea against tert-butyl hydroperoxide-induced oxidative damage. *Chem. Biol. Interact.* **2009**, *181*, 366–376, doi:10.1016/j.cbi.2009.07.017.
